# Marine Compounds, Mitochondria, and Malignancy: A Therapeutic Nexus

**DOI:** 10.3390/md20100625

**Published:** 2022-09-30

**Authors:** Sajad Fakhri, Sadaf Abdian, Seyed Zachariah Moradi, Blake E. Delgadillo, Carmela Fimognari, Anupam Bishayee

**Affiliations:** 1 Pharmaceutical Sciences Research Center, Health Institute, Kermanshah University of Medical Sciences, Kermanshah 6734667149, Iran; 2 Student Research Committee, Kermanshah University of Medical Sciences, Kermanshah 6714415153, Iran; 3 Medical Biology Research Center, Health Technology Institute, Kermanshah University of Medical Sciences, Kermanshah 6734667149, Iran; 4 College of Osteopathic Medicine, Lake Erie College of Osteopathic Medicine, Bradenton, FL 34211, USA; 5 Department for Life Quality Studies, University of Bologna, 47921 Rimini, Italy

**Keywords:** marine microorganisms, marine compounds, anticancer activities, mitochondria

## Abstract

The marine environment is important yet generally underexplored. It contains new sources of functional constituents that can affect various pathways in food processing, storage, and fortification. Bioactive secondary metabolites produced by marine microorganisms may have significant potential applications for humans. Various components isolated from disparate marine microorganisms, including fungi, microalgae, bacteria, and myxomycetes, showed considerable biological effects, such as anticancer, antioxidant, antiviral, antibacterial, and neuroprotective activities. Growing studies are revealing that potential anticancer effects of marine agents could be achieved through the modulation of several organelles. Mitochondria are known organelles that influence growth, differentiation, and death of cells via influencing the biosynthetic, bioenergetic, and various signaling pathways related to oxidative stress and cellular metabolism. Consequently, mitochondria play an essential role in tumorigenesis and cancer treatments by adapting to alterations in environmental and cellular conditions. The growing interest in marine-derived anticancer agents, combined with the development and progression of novel technology in the extraction and cultures of marine life, led to revelations of new compounds with meaningful pharmacological applications. This is the first critical review on marine-derived anticancer agents that have the potential for targeting mitochondrial function during tumorigenesis. This study aims to provide promising strategies in cancer prevention and treatment.

## 1. Introduction

Cancer is the uncontrolled growth of cells and formation of tumors caused by genetic changes, and in recent decades, it has been considered to be one of the major causes of death in the world [1,2]. Genetic disorders, exposure to variant physical and chemical agents, tobacco, radiation, infections, environmental pollutants, obesity, and diet are some of the important risk factors for cancer [1,2,3,4,5,6,7]. Cancerous cells have a variety of basic origins and pathologies, such as aberrant cell cycles, proliferation, apoptosis, and angiogenesis [8,9]. Based on the grade, type, and location of cancers, several important treatments include chemotherapy, hormonal therapy, radiation therapy, and surgery [10]. Despite these different therapeutic methods, the majority of initially responsive tumors obtain a multidrug resistance phenotype; thus, combination therapy has been preferred and used to treat various cancers [11,12]. These treatments play significant roles in destroying cancerous cells, but they do not have the potency to suppress all types of cancer, which led researchers to investigate more efficacious therapeutic options [13]. Over the last several decades, many approved anticancer agents have either been obtained from natural sources or their synthetics analogs have been developed [14].

In the creation of new functional foods, the marine ecosystem remains a goldmine of bioactive components. Natural organic substances derived from marine bio-resources, such as fatty acids, peptides, enzymes, polyethers, polysaccharides, and proteins, are some of the most potent, specialized, and bioactive chemicals used [15]. Due to the various qualities of marine derived compounds, these chemicals exhibit significant biological activity, including antiviral, anticarcinoma, anticoagulant, antibacterial, antioxidant, antibacterial, antihypertensive, antiproliferative, and antidiabetic potential [16,17,18,19]. Several previous reviews emphasize that these products are considered the key structures in developing novel therapeutic strategies for the management of cancers. They exhibit promising results in suppressing the proliferation, growth, and metastases of several cancerous cells via targeting multiple signaling pathways [15,20,21,22,23,24,25]. Cytarabine (Cytosar), trabectedin (Yondelis), eribulin mesylate (Halaven), and brentuximab vedotin (Acentris) are some of the known marine-based products that are approved for the treatment of several types of cancer [23,24,26]. The mitochondrial pathway is one of the most important signaling pathways in modulating a cell’s function. Mitochondria are one of the vital components of eukaryotic cells, and they play a significant role in producing energy and balancing cell survival/death. Additionally, mitochondria control oncogenic signaling, redox homeostasis, innate apoptosis, and immunity. Given the important role that mitochondria play, malfunction of these organelles is one of the most important causes of cell disturbance, and by modulating mitochondrial signaling pathways, researchers may discover new ways to prevent and treat cancers [27,28,29,30,31,32,33]. Previously, several natural products, with no focus on marine compounds, have been highlighted in targeting mitochondria in cancer [34]. Several marine agents have been highlighted in combating cancer through different mechanisms with no focus on mitochondria-related pathways [20,21,35,36]. The current comprehensive and critical review discusses the antineoplastic potential and mechanism of natural marine drugs for diminishing the metastasis, proliferation, and angiogenesis of several cancerous cell lines via modulating mitochondrial signaling pathways.

## 2. Mitochondria and Cancer: Biology and Cellular Signaling

Otto Warburg was the first to demonstrate that cancer cells, in the presence of oxygen, promote the fermentation of glucose, which may possibly be one of the underlying causes of cancer associated with mitochondrial respiration defects. In addition, researchers have concluded that genetic events that promote aberrant proliferation of cancer cells can also lead to alterations in biochemical metabolisms such as promoting aerobic glycolysis; although, this does not routinely impair mitochondrial function [37,38,39,40]. In most eukaryotic organisms, double-membrane-bound organelles, such as mitochondria, produce adenosine triphosphate (ATP) through aerobic respiration for use throughout the cell. Mitochondria control redox homeostasis, supply energy, influence oncogenic signaling, and regulate the cell cycle, apoptosis, and innate immunity. This mitochondrial biogenesis is often alternated in cancers. Mitochondria play an undeniable role in both lethal and vital functions in pathological and physiological conditions. In addition to the indispensable role of mitochondria in the production of energy and eukaryotic cell survival, mitochondria are important regulators of the intrinsic apoptotic pathway [37,38,39,40]. Numerous copies of the mitochondrial DNAs (mtDNAs) and nuclear DNA (nDNA)-encoded genes encompass the mitochondrial genome. In several cancers, mtDNA mutations have been found to affect mitochondrial metabolism, elevate tumorigenesis, and facilitate the adaptation of cancer cells to changing environmental conditions [40]. In addition, elimination of mtDNAs in cancer cells have been shown to diminish tumorigenicity via suppression of growth and progression of tumors. Thus, mitochondria have shown a meaningful multifunctional role in the progression of neoplasms and have made mitochondria an attractive target for therapeutic intervention [40]. 

Various mutations can affect mtDNA expression, and variant populations of mtDNA have been associated with cancer risk. African American women may have an increased risk of breast cancer risk if they have the macrohaplogroup N variant of the complex I subunit NADH dehydrogenase 3 gene (ND3) at nucleotide G10398A (resulting in an amino acid change of T114A). Endometrial cancer is associated with the 16519 T to C variant of the mtDNA control region [40,41,42]. In addition to mutations in several nDNA mitochondrial genes, the T6777C nucleotide variant of mtDNA’s cytochrome c (cyt. c) oxidase subunit 1 (MTCO1) has been associated with epithelial ovarian cancer [40]. Additionally, Chinese women with the variant C150 in the mtDNA control region have exhibited an elevated risk of cervical cancer and human papillomavirus infection [43]. Moreover, inhibition of mitochondrial oxidative phosphorylation (OXPHOS) is mentioned as another consequence of mtDNA mutations in many cancer cells [40]. In summary, two types of mutations in cancer cell mtDNA are important: mutations that promote the adaption of the cancer cells to changing bioenergetic environments and mutations that lead to stimulating neoplastic transformation via impairing OXPHOS [40].

Specific cancers have also been found to have mutations affecting mitochondrial enzymes encoded by nDNA and mutations directly affecting mtDNA. Succinate dehydrogenase (SDH) is one of these mitochondrial enzymes that may mutate [40,44]. Respiratory complex II (known as SDH) is an integral mitochondrial protein complex that transfers two electrons to coenzyme Q10 (CoQ) and oxidizes succinate to fumarate. SDHA-SDHD are the four main subunits that constitute this structure. SDH assembly factor 1 (SDHAF1) and SDHAF2 are two factors that are necessary for the formation and assembly of SDH. In preliminary research, SDHC, SDHB, and SDHD have been mentioned as mutated subunits of SDH in paragangliomas, and subsequent research confirmed the mutation in other parts, including SDHAF2 and SDHA [40,44,45]. Disruption of SDH elevated the cytosolic and mitochondrial levels of succinate, which suppressed the α-ketoglutarate dependent prolyl hydroxylase (PHDs). This resulted in the translocation of hypoxia-inducible factor 1α (HIF-1α) into the nucleus and its stabilization. Additionally, the inactivation of PHDs by reactive oxygen species (ROS) is another cause of HIF-1α stabilization; changing the energy metabolism from oxidative to glycolytic is the main result of these consecutive events. α-ketoglutarate-dependent dioxygenases, such as the TET family of 5-methylcytosine hydroxylase and Jumonji-C histone demethylases, have been shown to be suppressed by fumarate and succinate, which promotes the epigenetic dysregulation, DNA methylation, and alterations of histone [40,44,45]. 

Fumarate hydratase is another mitochondrial enzyme that converts fumarate to malate. Cells containing the mutant form of fumarate hydratase have markedly reduced levels of citrate and malate and elevated production of succinate and fumarate [37,40]. Several aggressive forms of renal carcinoma, uterine leiomyomas, and cutaneous leiomyomas have been associated with the fumarate hydratase gene mutations [46]. It has also been hypothesized that fumarate inhibits PHDs and stabilizes HIF-1α. Similarly, elevated levels of fumarate activate the stress response signaling that can be regulated by nuclear factor erythroid 2-related factor 2 (Nrf2). Degradation of Nrf2 by the ubiquitin E3 ligase cullin 3 and kelch-like ECH-associated protein 1 (KEAP1) keep its level low [37,40]. Additionally, excess fumarate leads to stabilized Nrf2 via inactivating KEAP1, which promotes turning on the nDNA stress-response genes, such as heme oxygenase 1 (HMOX1). This is the result of binding Nrf2 to antioxidant response elements (AREs). HMOX1 can increase the colony-forming capacity and activation of HMOX1 is an important marker of tumorigenesis [40,47].

Isocitrate dehydrogenase 1 (IDH1), a known enzyme encoded by the IDH1 gene, catalyzes the oxidative decarboxylation of isocitrate to 2-oxoglutarate (α-ketoglutarate) [48]. Several types of cancer, including gliomas, acute myeloid leukemia, astrocytoma, and chondromas, have been found to harbor heterozygous missense mutations in the nicotinamide adenine dinucleotide phosphate (NADP^+^)-dependent IDH enzymes, mitochondrial IDH2 enzymes, and cytosolic IDH1 enzymes [40,48,49]. IDH1-R132, IDH2-R140, and IDH2-R172 are common IDH mutations in gliomas. Mutant IDH2-R140 is the most common reported IDH mutation in acute myeloid leukemia [40,48,49,50]. IDH1 and IDH2, unlike IDH3, decrease the NADP^+^ instead of NAD^+^ in catalyzing the decarboxylation of isocitrate to α-ketoglutarate. The potential of NADPH to supply sufficient energy to perform the reaction led to reversibility of the NADP+-dependent reaction. Mutant IDH2-R172 and IDH1-R132 utilize NADPH to diminish α-ketoglutarate and convert it to R(−)-2-hydroxyglutarate, which is considered an “oncometabolite” [40]. Excess R(−)-2-hydroxyglutarate activates PHD1 and PHD2 resulting in reduced HIF-1α. R(−)-2-hydroxyglutarate is a potent inhibitor of JMJD2D, JMJD2C, and JMJD2A (α-ketoglutarate dependent Jumonji-C domain histone Nε-lysine demethylases) and is associated with interfering transcription patterns and methylating cellular genomes. Mutant IDH1 promotes enhancing trimethylation of histone H3 at lysine 9. Consequently, elevated methylation of DNA is found within the CpG island. In glioblastomas, extreme CpG island methylation can also be found [40,51].

**Figure 1:**
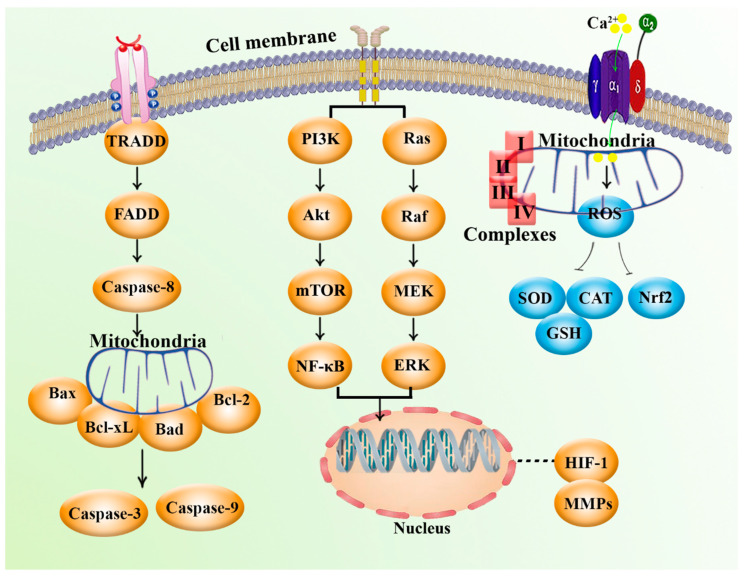
The major mediatory role of mitochondria and related signaling mediators in cancer.

Glioblastomas cells with mutant IDH1, IDH2, and CpG island methylator phenotype (CIMP) showed remarkable alteration in the expression of variant genes, including WNT, epidermal growth factor receptor (EGFR), transforming growth factor-β (TGFβ), RAS, and angiogenesis-related genes. Bioenergetic changes could accompany such alteration in chromatin structure, as the WNT pathway regulates mitochondrial energy metabolism [40,52,53]. In addition, Mn superoxide dismutase (MnSOD) dismutates mitochondrial matrix superoxide into H_2_O_2_, and cytosolic and intermembrane Cu/ZnSOD (SOD1) dismutate intermembrane-space superoxide [54,55]. Glutathione and glutathione peroxidases reduce H_2_O_2_ and other peroxides generated by mitochondria using NADPH. Mitochondrial ROS are potent mitogens and important signaling molecules, and the excess production of mitochondrial ROS can promote apoptosis or necrosis [40]. Furthermore, NADPH functions as a regulator of protein function through the reduction of thiols (-SH). It is possible for the bifunctional AP endonucleases 1 (APE1) protein to decrease the FOS–JUN sulfenic acids with NADPH via the thioredoxin 1. APE1 can regulate nuclear factor-κB (NF-κB), Nrf2, glucocorticoid receptor (GR), p53, NLR family pyrin domain containing 3 (NLRP3), and HIF-1α via decreasing cysteine residues in these proteins [40,56] (Figure 1).

Moreover, mutation of the promyelocytic leukemia gene or reduction of the potential of mitochondrial membrane diminishes the Ca^2+^ uptake capacity of mitochondria, which leads to the suppression of the activation of the intrinsic (mitochondrial) apoptosis pathway [40]. Moreover, decreased Ca^2+^ retention in mitochondria leads to elevated concentrations of cytosolic Ca^2+^. This condition promotes mitochondrial retrograde signaling via activation of IκBβ-dependent NF-κB, calcineurin, and enhanceosome-driven transcription, as well as elevation of the metastatic potential [40] (Figure 1).

Furthermore, one of the most general alterations found in cancer is the activation of the phosphoinositide 3-kinase (PI3K)–phosphatase and tensin homolog (PTEN)–protein kinase B (Akt) signaling pathway, which results after the shift from oxidative to glycolytic metabolism [57]. Similarly, p53, a tumor suppressor protein, can initiate apoptosis and promote suppression of cell growth. The limitation of energy can lead to induced phosphorylation of p53 by AMP-activated protein kinase (AMPK), which facilitates the activating checkpoints of the cell cycle. In addition, Akt and phosphoglycerate mutase can be negatively regulated by p53. This leads to the upregulation of OXPHOS complex IV and inhibition of glycolysis via the induction of the cyt. c oxidase (SCO2) [40,54,57]. Under low oxygen tension, the transcription factor HIF-1 promotes glycolysis via inhibition of mitochondrial function, which induces the upregulation of the expression of genes encoding glycolytic proteins, glucose transporters, as well as angiogenesis-related factors, including vascular endothelial growth factor (VEGF) and erythropoietin [58,59]. In addition, HIF-1 can affect mitochondria via targeting several mechanisms and parameters, such as inducing PDHK1 and cyt. c oxidase subunit 4 isoform 2 (COX4-2), inhibiting MYC signaling, and upregulating the transcription of miR-210 [40,58,59] (Figure 1). 

In summary, the presence of mutations in mtDNA and nDNA, disturbances in the mitochondrial metabolism-related enzymes (such as IDH1, IDH2, SDH, and fumarate hydratase), activation of variant oncogenes and signaling pathways with main tumorigenesis potential (including PI3K–PTEN–Akt, HIF-1α, WNT, p53, and angiogenesis-related signaling), alterations of mitochondrial metabolism, cellular redox status, increased production of ROS, reduced mitochondrial Ca^2+^ retention, and mitochondrial membrane potential (MMP) are the main factors and signaling pathways that affect mitochondrial function and have been found in various cancers (Figure 1).

## 3. Synthetic Agents and Candidate Phytocompounds in Modulation of Cancer through the Regulation of Mitochondria Function

Nature is one of the main sources of active therapeutic agents, chemical compounds, and drugs with a wide variety of pharmacological effects, including antitumor, cardioprotective, antiviral, neuroprotective, anti-inflammatory, and antibacterial activities [60,61,62,63,64,65,66,67,68,69,70,71]. In recent decades, various mitochondrial-targeted components with significant anticarcinoma potential have been identified and obtained from natural compounds. 

**Figure 2:**
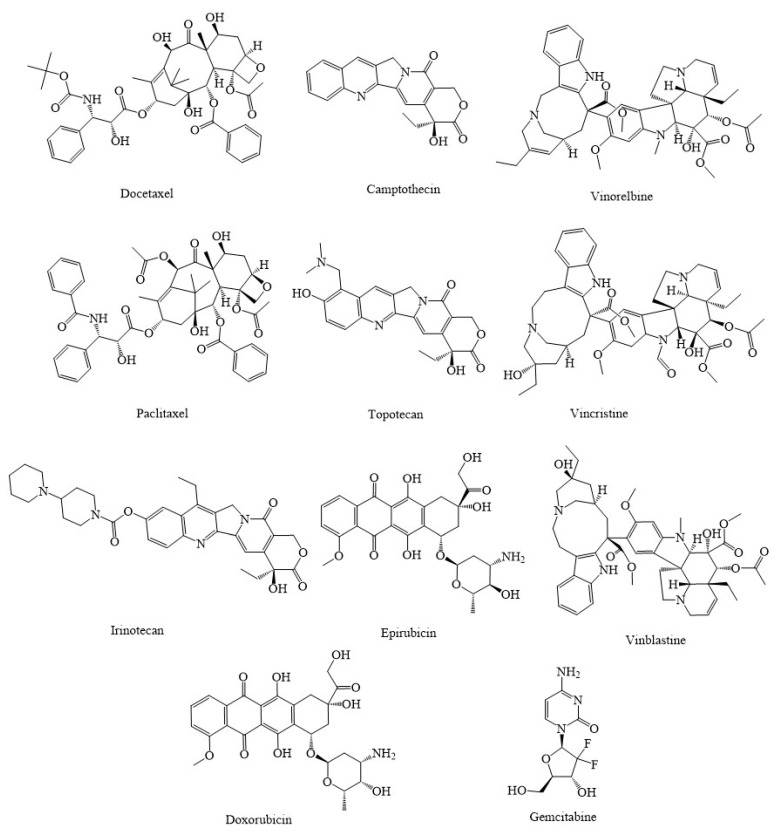
Chemical structures of several approved anticancer compounds combating cancers through different mitochondria-related signaling pathways.

Paclitaxel (Taxol) and the analogs cabazitaxel (Jevtana) and docetaxel (Taxotere), camptothecin, vinorelbine (Navelbine), topotecan (Hycamtin), vincristine (Oncovin), irinotecan (Camptosar), epirubicin (Ellence), and vinblastine (Velban) (Figure 2) are some of the synthetic and semisynthetic analogs of antitumor drugs derived from plants that are routinely used in the treatment of various types of cancers through mitochondria [60]. Doxorubicin-loaded triphenylphosphine (TPP)-conjugated chitosan nanoparticles (NPs) (Dox-loaded TPP-NPs) played an essential role in controlling the apoptotic signaling pathway by lowering MMP, enhancing ROS, Bcl-2-associated X (Bax), and stimulating the release of cyt. c, which consequently led to activation of caspase-9 and caspase-3 [72]. The combination of paclitaxel and doxorubicin co-loaded liposomes (PD-LPs) has been demonstrated to be a useful strategy for clinical cancer medication with minimal toxicity and treatment failure. This was accomplished while boosting therapeutic potential in SMMC-7721 cells by modulating apoptotic pathways via increased production of Bax and caspase-3 and reduced levels of Bcl-2; thus, it can play an important role in the treatment of hepatocellular carcinoma [73]. 

**Figure 3:**
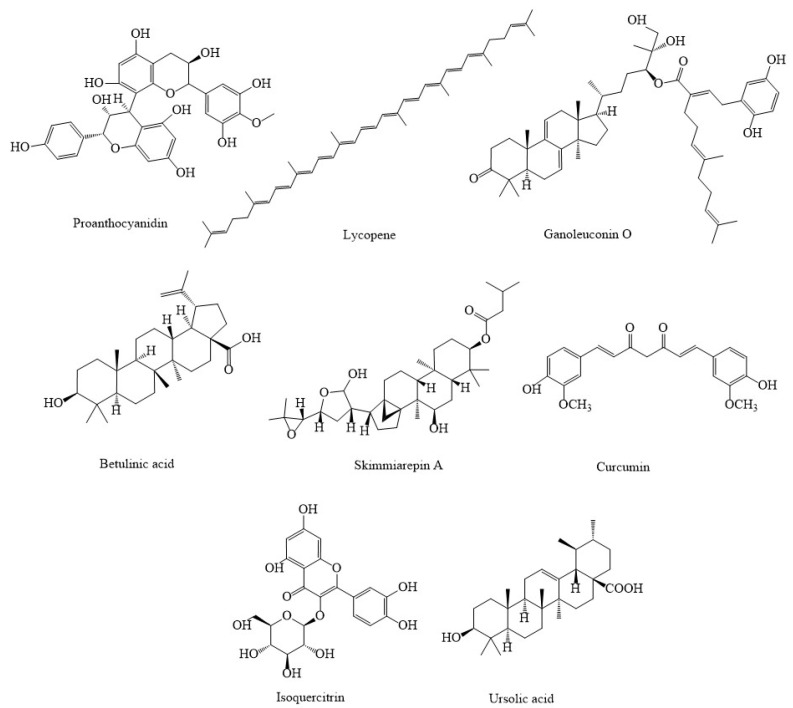
Chemical structures of some candidate non-marine natural products combating cancers through different mitochondria-related signaling pathway.

Proanthocyanidin (Figure 3) combined with chitosan nanoparticles (CS-PAC-AgNPs) has been shown to be effective in the treatment of colorectal cancer by modulating mitochondria function. CS-PAC-AgNPs can activate the release of cyt. c, induce the activation of caspase-9 and caspase-3, inhibit cancerous cellular proliferation, and suppress the formation of antiapoptotic proteins in HT-29 cells [74]. Lycopene, a tetraterpene present in tomatoes and several foods, is the most abundant carotenoid accumulating in prostate tissues and has been found to have antineoplastic properties in prostate cancer. This antioxidant agent can regulate several cell signaling pathways that can lead to a decrease in the mitochondrial transmembrane potential, trigger the production of mitochondrial cyt. c, and induce apoptosis in LNCaP human prostate cancer cells [75,76]. Ganoleuconin O, a terpenoid compound obtained from *Ganoderma* *leucocontextum*, has shown anticancer effects in liver cancer cell line Huh-7.5 by inducing apoptosis pathway via increasing p53, Bax, caspase-9, and release of cyt. c while reducing the rate of ATP, Bcl-2; all of these lead to mitochondrial dysfunction [77]. Betulinic acid, another terpenoid compound isolated from *Betula alba,* has shown considerable anticancer activities in HeLa, PC12, and ACHN cancer cells by inducing mitochondrial dysfunction via enhancing the rate of ROS, caspase-3, caspase-9, Bax, and reducing Bcl-2 [77]. Skimmiarepin A and skimmiarepin C, two compounds derived from the Bael tree (*Aegle marmelos*), have shown antimalignancy impacts in human breast tumor T47D cells through blocking HIF-1 stimulation and silencing cellular respiration by selectively blocking the mitochondrial electron transport chain at complex I; thus, it may be a promising treatment for breast cancer [78]. Curcumin, a polyphenol effective factor isolated from the rhizome of *Curcuma longa*, has shown anticarcinogenic effect in HepG2 cells through stimulating apoptosis in mitochondria by increasing the production of proapoptotic protein Bax, decreasing the formation of antiapoptotic protein Bcl-2, promoting cyt. c release, and increasing the level of caspase-3 [79]. Isoquercitrin is a flavone compound that can diminish the growth of MDA-MB-231 by causing mitochondrial dysfunction via decreasing the levels and activities of MMP, Bcl-2, and increasing Bax in relation to mitochondria [77]. 

The role of natural and semisynthetic agents in the prevention, treatment, and decreasing the drug resistance in cancer is quite clear. Natural and synthetic agents apply these substantial pharmacological effects by affecting several signaling pathways. Natural products potentiate the interference with variant anticancer signals related to mitochondrial activities, including VEGF, p53, Bax/Bcl-2, caspases, cyt. c, HIF-1, MMP, and ROS-related pathways. Table 1 summarizes various anticancer phytochemicals and synthetic agents that interfere with the mitochondrial-related pathways.

## 4. Marine Compounds Suppress Cancer through the Modulation of Mitochondria Function

The marine environment has long been a significant supplier of natural resources. With its vast expanse that covers 70% of the planet’s surface, it has the ability to hold a variety of different creatures [85,86]. Marine life are well-known makers of new small agents with distinct chemical structures and potentially beneficial biological effects [87]. These small compounds, known as secondary metabolites, and other various compounds are small molecules that are major sources of active ingredients and novel medications, which are abundant in nature [77]. Many of these marine-derived natural products have beneficial roles in the treatment of various types of cancer, so they can introduce a new way to pave the road against cancer [16,17,20,21,35,36]. 

**Figure 4:**
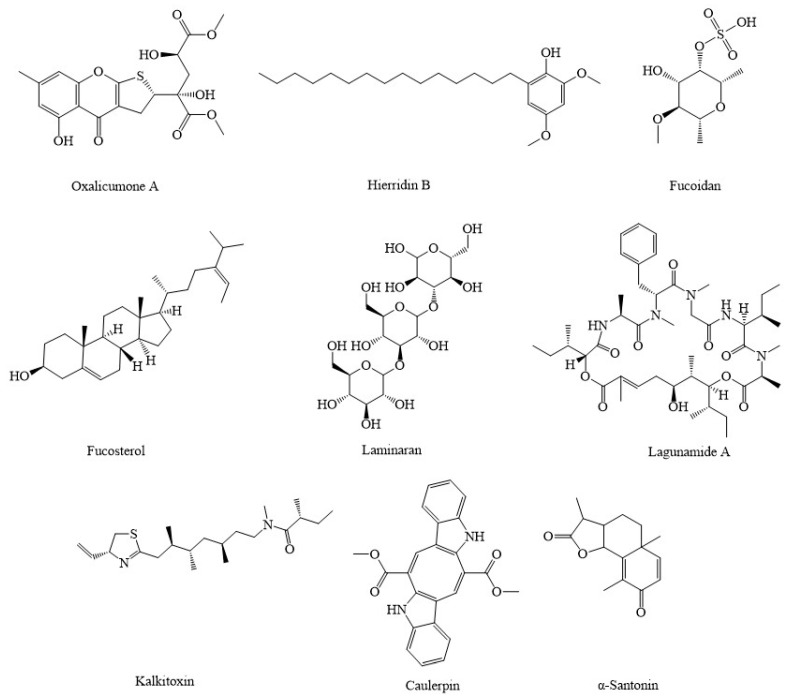
Chemical structures of candidate marine compounds combating cancer through mitochondrial pathway.

Experiments with oxalicumone A (Figure 4), an organic chemical compound obtained from marine sources fungus *Penicillium oxalicum*, have proven that it is a powerful bioactive antitumor agent for a range of human carcinomas [88]. An immediate decrease in the rate of ATP production has been seen following attenuation of mitochondria function with oxalicumone A. This was followed by mitochondrial permeability transition pore (MPTP) activation and mitochondria enlargement, vacuolation, and reduced matrix density [88]. As another marine agent, hierridin B, is obtained from a marine cyanobacterium (*Cyanobium* sp.), which has recently been identified as a potential resource for the extraction and purification of chemicals having therapeutic properties, particularly as anticancer drugs [89]. Significant alterations in voltage-dependent anion channel-1 (VDAC1, a mitochondrial protein in the outer mitochondrial membrane that causes a central pore and is considered a major protein for mitochondria-mediated apoptosis) were caused by hierridin B on colon adenocarcinoma cell line HT-29. There was a drop in mRNA and protein expression of VDAC1, and the activity of mitochondrial was reduced [89]. As marine compounds isolated from algae, spatane diterpenoid, 5(*R*), 19-diacetoxy-15,18 (*R* and *S*), dihydro spata-13, 16 (E)-diene (DDSD), which were isolated from the brown marine algae *Stoechospermum marginatum*, were previously shown to have suppressive effects on various human cancer cell lines and murine melanoma cells [90]. DDSD triggered the production of ROS, which resulted in a change in the Bax/Bcl-2 ratio, which disturbed the inner mitochondrial transmembrane potential. This caused the redistribution of Cyt c to the cytoplasm and the stimulation of the caspase-mediated apoptotic pathway [90]. Another marine agent obtained from algae, fucoidan, which is isolated from brown algae, has been found to exhibit significant anticancer potential [91]. Cell death-inducing pathways, such as the c-Jun N-terminal kinase (JNK), p38, and extracellular signal-regulated kinases (ERK) 1/2, were activated in MCF-7 cells after exposure to fucoidan essence. These pathways are implicated in mechanisms that cause cell death [92]. The production of antiapoptotic proteins Bcl-2 and Bcl-xL was reduced in response to fucoidan essence therapy, although proapoptotic proteins Bax and Bad were slightly increased [92]. Fucoidan stimulated Cyt c production, and it significantly suppressed antiapoptotic proteins, such as Bcl-2 and Bcl-xL in MDA-MB-231 breast cancer cells [91,92]. Additionally, fucoidan led to decreasing Bax/Bcl-2 levels, which enhanced the production of ROS and Bax-to-Bcl-2 ratio in SMMC-7721 cells [93]. In this line, fucosterol (24-ethylidene cholesterol) is one of the phytosterols found in brown algae, and it exerts a variety of biological activities, notably anticancer capabilities. It can suppress the PI3K/Akt pathway and show apoptotic properties [94]. Fucosterol caused ROS production in HeLa cells and mitochondrial malfunction in ES2 and OV90 cells, activated caspase-3 and caspase-9, released cyt. c, and caused the impairment of MMP [17,94]. Brown algae are also a source of the β-glucan-like laminarin, which has several beneficial effects, including anti-inflammatory, antioxidant, and anticancer properties. Laminarin suppressed the PI3K/mitogen-activated protein kinase (MAPK), which modulated dysregulated pathways in ovarian cancer cells (ES2 and OV90). In addition, it had a continuous weakening effect on cell growth [95]. In ES2 and OV90 cells, laminarin caused MMP degradation, which led to an elevation in intracellular calcium concentrations, and it caused ROS production, which induced the production of Cyt c and enhanced the destruction of DNA [95]. Laminarin also increased ROS production, which stimulated MPTP, increased Ca^2+^, cyt. c, caspase-3, and Bax concentration, and decreased the MMP in LOVO cells [96]. Desmethylxestospongin B, also known as dmXe B, is a complex marine natural compound that displays unique capabilities as a calcium pathway modulator and has downstream impacts in the mitochondrial metabolism of cancer cells [97]. Through the suppression of inositol-1,4,5-triphosphate receptors, xestospongin B was able to exert an effect on mitochondrial metabolism. This was accomplished via regulating calcium communication between the endoplasmic reticulum (ER) and mitochondria that resulted in a decrease in mitochondrial respiration, which in turn caused the stimulation of AMPK and autophagy [97]. The sulfated polysaccharide (EI-SP), a compound that is present in marine algae and isolated from *Enteromorpha intestinalis,* has been demonstrated to have antioxidant, antineoplastic, and anti-inflammatory properties [98]. EI-SP elevated the amount of the proapoptotic Bax protein while it reduced the amount of the antiapoptotic protein Bcl-2 in human hepatoma hepG2 cells. It was concluded that a sulfated polysaccharide, EI-SP, exhibited anticancer properties. Additionally, it increased the level of cyt. c, which resulted in increased activity of caspase-9 [98]. Similar in source, thyrsiferol, extracted from the tropical sea red alga *Laurencia thyrsifera* J.Agardh, was discovered to have high anticancer potential against multiple tumor cells through different signaling pathways [99]. Thyrsiferol, which was also separated from the tropical marine red alga *Laurencia thyrsifera* J.Agardh, was found to suppress HIF-1 stimulation in T47D human breast tumor cells, as well as selectively block mitochondrial respiration at complex I and oxygen utilization in a concentration-dependent way [99]. Caulerpin, a secondary metabolite derived from the marine invasive green algae *Caulerpa cylindracea*, has been demonstrated to have antineoplastic impacts in many cancerous cell lines via multiple signaling pathways [100]. Caulerpin has been shown to block the function of complex II in the mitochondria respiratory system and can be beneficial in the treatment of human ovarian carcinoma [100]. *Chlorella sorokiniana*, a species of green algae commonly consumed as a food supplement in East-Asian countries, has shown a useful role on NSCLC by modulating apoptotic pathways [101]. *Chlorella sorokiniana* can enhance the rate of caspase-3, caspase-9, and Bax, reduce MMP, which induces the release of cyt. c, lower the rate of Bcl-2, and suppress the proliferation of non-small cell lung cancer [101].

Lagunamides A is a cyclic depsipeptide initially identified from the marine cyanobacterium *Lyngbya majuscule*. Lagunamides A has been shown to possess exceptional growth-suppressive properties against non-small-cell lung carcinoma [NSCLC] (A549), cervical carcinoma cells (HeLa), hepatocellular carcinoma cell (HepG2), BEL-7404, colorectal carcinoma cells (HCT116), and osteosarcoma (U2OS) cancer cells [102]. Additionally, lagunamide A enhanced the production of ROS, which caused cell death, and there was also a reduction in the MMP as a result of lagunamide A therapy [102]. As another peptide type marine compound, irciniastatin A (psymberin), a pederin-type natural compound isolated from a marine sponge *Ircinia ramosa*, showed particularly anticancer effects against human leukemia Jurkat cells [16]. Irciniastatin A is a strong inhibitor of protein production, and through a mitochondrial-mediated process, it caused sustained stimulation of the stress-activated protein kinases JNK and p38 by modulating production of ROS in mitochondria [16]. MSPs are marine antimicrobial peptides and natural compounds isolated from *Nile tilapia* (*Oreochromis niloticus*). MSPs have been shown to exert antitumor roles in the treatment of several cancers through different signaling pathways, especially by inducing apoptotic pathways [103]. MSP-4-peptide caused a decrease in the production of antiapoptotic proteins like Bcl-2 and increased proapoptotic proteins like Bax and Bid, releasing cyt. c, and increased caspase-9 and caspase-3 in mitochondria in MG63 cells in in vitro models of human osteosarcoma [103]

Conotoxins and conus textile, derived from marine cone snails in Iran’s southern seas, have been demonstrated to exhibit antitumor properties in U87MG human glioma cells in mitochondrial-related pathways [104]. *Conus textile*, a carnivorous species, has been shown to induce the activity of caspase-3, caspase-9, and Bax/Bcl-2 levels, and enhance the production of cyt. c and ROS in U87MG cells [104]. Complexes II and III were severely suppressed by 3,4,5-tribromo-2-(20,40-dibromophenoxy)-phenol, whereas complexes I and V were very mildly suppressed [105]. 

A solitary marine tunicate of the ascidian class in tropical seas, *Phallusia nigra* compounds generated considerable ROS production in skin mitochondria from the melanoma group, but they did not in the control group. *Phallusia nigra*, a unique marine tunicate with significant antitumor activities, exhibits high promise and practicality as a source of anticancer medications [106]. Additionally, *Phallusia nigra* compounds enhanced cyt. c released and reduced mitochondria membrane potential (MMP) in Albino/Wistar rats [106]. Aplidin (plitidepsin), a cyclic depsipeptide derived from the marine tunicate *Aplidium albicans* and now produced synthetically, has been demonstrated as having antineoplastic properties in many hematological and solid cancers through the apoptotic process and several signaling pathways, such as p38 and MAPK [107]. Aplidin has been demonstrated as having positive effects on leukemia lymphoma models in vitro and in vivo by modifying mitochondria through producing ROS and decreasing MMP and ATP concentrations [107]. Lipopeptide kalkitoxin, isolated from the *Lyngbya majuscula* marine cyanobacterium (often reported as *Moorea producens*), prevented hypoxic HIF-1 induction, reduced the quantity of hypoxia-induced secreted VEGF protein, and prevented tumor angiogenesis, as well as regulated mitochondrial respiration in tumor cells by blocking electron transport chain complex I [108]. 

Methyl 5-[(1E,5E)-2,6-Dimethyl octa-1,5,7-trienyl] furan-3-carboxylate (MDTFC), a furano-sesquiterpene bioactive component from a soft coral, has shown to induce apoptosis in THP-1 (human monocytoid) cells via reducing caspase-3, caspase-9, enhancing the rate of Bax/Bcl-2, releasing cyt. c, and decreasing MMP, which may make it an effective treatment in cancer [109]. 2-ethoxycarbonyl-2-β-hydroxy-A-nor-cholest-5-ene-4one (ECHC), a marine steroid compound derived from butanol extracts of the scleractinian coral *Acropora formosa*, may be useful in the treatment of lung cancer [110]. ECHC from scleractinian coral *Acropora formosa* can enhance the rate of ROS, cyt. c, Bax, tumor suppressor p53, and reduce the production of antiapoptotic factors, such as tumor necrosis factor-α (TNF-α), interleukin-8 (IL-8), Bcl-2, MMP-2, and MMP-9 in A549 human non-small cell lung cancer cell lines [110]. 

The *Turbo coronatus*, a Persian gulf marine mollusk that is a member of the *Turbinidae* family, was found to have antitumor and apoptotic impacts in epithelial ovarian cancer cells [111]. It has shown to inhibit SDH function, enhance the production of ROS and the release of cyt. c, reduce MMP, and enhance the activity of caspase-3 in mitochondria of human EOC cells [111]. Isolated from other marine sources, mansouramycin C, a cytotoxic isoquinolinequinone gained from a marine streptomycete, has been shown to have anticancer activity in tumoral cells via targeting different signaling pathways [112]. Mansouramycin C enhanced the production of ROS in cancerous cells, reduced the rate of ATP and MMP, and stimulated mitochondrial swelling via the opening of MPTP in A549 cells; thus, it may become a hopeful candidate for the treatment of cancer [112].

**Figure 5:**
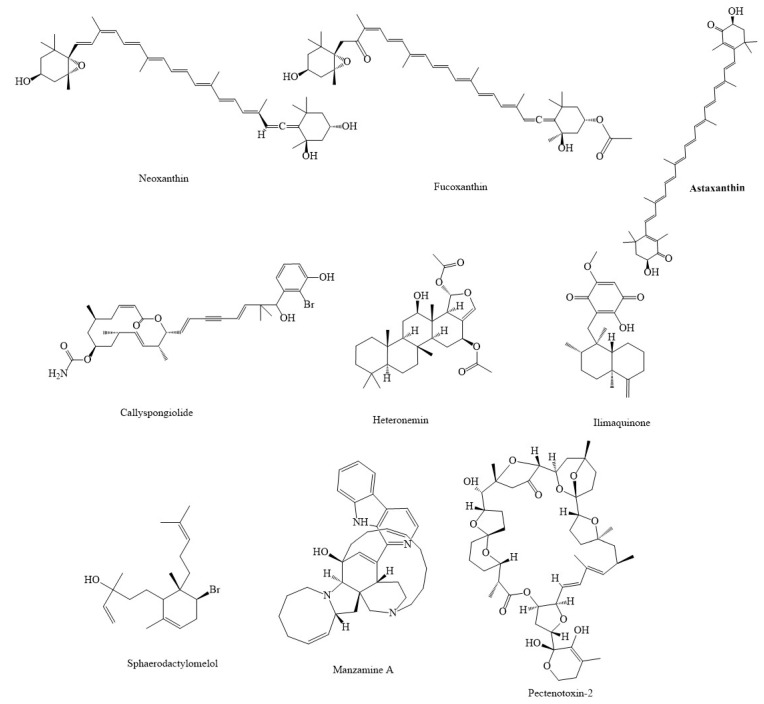
Chemical structures of other marine compounds combating cancer through mitochondrial pathway.

Highly polar xanthophylls of 9′-cis-neoxanthin and fucoxanthin (Figure 5), two carotenoids, have been found to diminish the survival of human prostate cancer cells by promoting apoptosis [113], reducing the rate of ATP synthesis, and enhancing the production of Bax and cyt. c in mitochondria [114]. Fucoxanthin has been shown to decrease cell cycle development in human neuroblastoma cells while causing cell death in HL-60 and human colon cancer cells [115]. Additionally, fucoxanthin enhanced ROS and reduced the levels and activity of MMP, SOD, catalase, and glutathione in oral squamous carcinoma (KB) cells [116]. Neoxanthin aggregation in mitochondria resulted in the reduction of MMP, which led to the production of cyt. c, apoptosis-inducing factor (AIF), and induced the activation of apoptotic processes [115]. Astaxanthin (3,3-dihydroxy-β,β′-carotene-4,4-dione), a carotenoid recognized for its powerful antioxidant capacity, has shown to have anticarcinoma properties in numerous cancer cell types via various signaling pathways. In this way, astaxanthin can enhance the level of proapoptotic proteins like Bax/Bad, reduce antiapoptotic proteins, such as Bcl-2, and can decrease the rate of ROS in glioblastoma multiforme cells [117]. Additionally, cellulose nanocrystals/nanofibrils loaded with astaxanthin nanoemulsion enhanced the production of ROS in L929 and NIH3T3 cells as in vitro models of skin cancer [118]. 

*Arca inflata* is a classical Chinese marine drug with a variety of therapeutic applications, including cancer therapy. It contains a potent peptide compound known as p6, which has been shown to have anticancer activities in human colorectal cancer cells via interfering with mitochondria-related pathways [119]. *Acta inflata* enhanced the rate of production of ROS, and Ca^2+^, released cyt. c, decreased MMP, and promoted the activity of caspase-3 in colorectal cancer cells, which causes apoptosis and led to mitochondrial malfunction. Additionally, p6 blocked the growth of colorectal cancer cells [119]. CS5931, a novel marine polypeptide extracted from sea squirt *Ciona savignyi*, has been shown to enhance the production of caspase-3, caspase-9, cyt. c, and Bax. It also interrupted MMP and suppressed the growth of HCT-8 colon cancer cells [120]. CS5931 has demonstrated strong cytotoxicity against several cancerous cells by intervening on caspase-3, caspase-9, cyt. c, Bax, and MMP [120]. Lamellarin D, a powerful marine alkaloid isolated from the marine mollusk *Lamellaria,* has been shown to block topoisomerase I and other signaling pathways, resulting in cytotoxic and antitumoral impacts in leukemia cells (p388) [121,122,123]. Lamellarin D increased the rate of Bax, stimulated the activity of caspase-3, caspase-9, and decreased Bcl-2, which led to mitochondrial malfunction and apoptosis in p388 leukemia cells [121,124]. 

GLP, a glycoprotein from *Codium decorticatum*, has been shown to stimulate the release of cyt. c and enhance the rate of ROS production, increase caspase-3, caspase-9, and decrease MMP in MDA-MB-231 cells [125]. It demonstrated cytotoxic impacts in human MDA-MB-231 cells via the apoptosis pathway. It reduced the growth of cancer cells and caused mitochondrial dysfunction [125]. In A549 lung cancer cells, the marine mangrove plant *Avicennia marina* combined with Ag NPs demonstrated an antineoplastic impact by enhancing the formation of ROS, destroying the mitochondrial membrane, and increasing the level of activated caspases, which resulted in apoptosis [126]. Pterocellin A, an alkaloid metabolite derived from the marine bryozoan *Pterocella vesiculosa*, has been shown to have antitumor effects against the human cervical cancer cell line HeLa. It has the potential to cause cytotoxic effects via inducing apoptosis and disrupting the morphology of cervical carcinoma cells (HeLa) [127]. Pterocellin A has been shown to induce the activation of caspase-3, and as a result of nucleus condensation, cause shrinkage, which led to the death of the HeLa cells [127]. Callyspongiolide decreased MMP and level of complex I and II, which decreased the activity of HIF-1A in mitochondria of various kinds of cancerous cell lines [128]. 

Heteronemin, a marine organic product obtained from *Hippospongia* sp., has been shown to defend against various cancerous cell lines by modulating the apoptosis pathway [129]. Heteronemin enhanced the rate of ROS, Bax, SOD2, and caspase-8 production, which decreased the level of Bcl-2 and SOD1 in human hepatocellular carcinoma cells. In addition, heteronemin led to mitochondrial dysfunction [129]. Brominated diterpene sphaerodactylomelol, derived from the red algae *Sphaerococcus coronopifolius* Stackhouse, has shown antitumor properties in breast cancer cells (MCF-7) via modulating the apoptosis signaling pathway [130]. Sphaerodactylomelol enhanced the rate of ROS, H_2_O_2_ production, caspase-3, caspase-9, Bax, and cyt. c, while decreasing antiapoptotic proteins, such as Bcl-2, and weakening MMP [130]. 

The marine compounds containing quinone moiety are known to target mitochondria. Ilimaquinone, a sesquiterpene quinone isolated from marine sponges (*Halichondria* sp.), has been shown to have antitumor effects on MCF-7 and MDA-MB-231 breast cancer cells by modulating various signaling pathways, particularly the mitochondrial pathway [131]. Ilimaquinone increased the rate of caspase-3, caspase-9, ROS, and MMP loss, which led to mitochondrial dysfunction in human breast cancer cells [131]. Another marine-derived polycyclic quinone-type metabolite, xestoquinone, decreased the growth of leukemia cancer cells, through multiple signaling mechanisms, including ROS-induced suppression of heat shock protein-90 [132]. A novel derivative of 1,4-naphthoquinone analogs isolated from the sea urchin pigments spinochromes, 6-S-(1,4-naphthoquinone-2-yl)-d-glucose chimera molecules, also showed anticancer potentials against human prostate cancer PC3 cells. The underlying mechanism of action included mitochondria membrane permeabilization, ROS upregulation, and release of AIF and cyt. c to the cytoplasm. This process led to the activation of caspase-9 and caspase-3, PARP cleavage, and apoptosis-like cell death [133]. Several other sesquiterpene quinones isolated from a marine sponge *Hippospongia* sp. Also suppressed the maturation of starfish oocytes and induced cell cycle arrest in HepG2 hepatocellular carcinoma cells [134]. 

Fraction 2.1, a polysaccharide gained from marine clam *Donax variabilis*, has shown anticarcinoma effects in A549 cells via inducing mitochondria malfunction by enhancing the level of Bax, activity of caspase-3, caspase-9, and stimulating the release of cyt. c while reducing Bcl-2 [135]. Manzamine A, an alkaloid obtained from sponges of the genera *Haliclona* sp., *Xestospongia* sp., and *Pellina* sp., has been shown to have the ability to destroy HCT116 cells by causing mitochondrial dysfunction by MMP loss, enhance the activity of caspase-3, caspase-7, induce the release of cyt. c, and decrease the level of Bcl-2 and Bcl-xL [136]. Aplysinopsins, a marine indole alkaloid isolated from genera of sponges and scleractinian corals, has exhibited antiproliferative effects on in K562 cells. EE-84, one of the analogs of aplysinopsins, reduced cell proliferation, intake of oxygen, level of antiapoptotic protein, Bcl-2, and caused MMP loss in K562 cells [137]. Another natural product from the marine sponge and fungus, *Aspergillus* sp., 18B-15-3, has exhibited anticarcinoma properties in pancreatic cancer. This substance exerted anticarcinoma effects by interfering with multiple pathways, especially the modulation of mitochondrial pathways [138]. 18B-15-3, marine-derived fungus *Aspergillus* sp., isolated from a marine sponge collected on Pramuka Island, suppressed complex III in the mitochondrial electron transport chain and reduced MMP, which blocked the normal function of mitochondria. Additionally, it blocked the intake of oxygen in pancreatic cancer cells [138]. 3,5-dibromo-2-(20,40-dibromophenoxy)-pheno and 3,4,5-tribromo-2-(20,40-dibromophenoxy)-pheno (20,40-dibromophenoxy)-phenol, other marine agents extracted from the marine sponge *Dysidea* sp., have been found to have anticancer effects in pancreatic cancer (PANC-1 cells) via increasing the levels of phosphorylated Akt [105]. 

Holothuroids (Holothuroidea class) are marine invertebrates which live in deep seas and are thought to be the origin of major drugs, such as anticancer medicines. Compounds obtained from sea cucumber (*H. parva*) and sponge (*H. oculata*) showed antitumor actions on liver mitochondria isolated from hepatocellular cancer in vivo [139]. *H. parva* and sponge *H. oculata* increased ROS concentration, decreased MMP, enhanced cyt. c release, and induced caspase-3 activity in the mitochondria, which had antineoplastic impacts in cancerous hepatocytes in male Sprague-Dawley rats [139]. *Ciona savignyi*, a marine agent containing a powerful polypeptide called *Callyspongiolide*, is an organic marine macrolide compound that exerted antimalignancy properties by inducing cell death in cancer [128]. Lipophilic 2,5-disubstituted pyrroles from the marine sponge *Mycale sp*. have been demonstrated to have antitumor actions in hypoxic conditions, which lead to the formation of solid tumors via mitochondria-related pathways [140]. Lipophilic 2,5-disubstituted pyrroles were shown to have antimalignancy effects in a human breast tumor T47D cell-based reporter assay by suppressing the function of HIF-1. This heterodimer protein was involved in the production of factors that are linked to angiogenesis, glucose metabolism, cell proliferation/survival, and invasion/metastasis. This was achieved via blocking the mitochondrial respiration through suppressing complex I and complex III, which generated ROS [140]. 

Urupocidin A, which is derived from marine sponges, is a bicyclic guanidine alkaloid that has been shown to have biological effects, particularly anticancer effects [87]. Urupocidin A was shown to increase the production of ROS, which was caused by a disruption in the integrity of mitochondria and MMP. It also reduced the expression of Bcl-2, which caused mitochondria to release various proteins which have antiapoptotic effects on prostate cancer cells [87]. The extraction of papuamine from *Haliclona* sp., a marine sponge, has been shown to exhibit anticancer effects in MCF-7 cells via regulating autophagy and mitochondrial dysfunction [141]. A furanoterpenoid isolated from irciformonin B found in a marine sponge, 10-acetylirciformonin B blocked Bcl-2 and Bcl-xL, enhanced Bax, stimulated the release of cyt. c, and increased the levels of ROS in mitochondria [142]. 10-acetylirciformonin B, has been shown to exhibit antitumor properties in leukemia HL 60 cells via promoting DNA destruction and apoptosis through several signaling pathways. 10-acetylirciformonin B dramatically reduced caspase-3 and caspase-9 activity and decreased topoisomerase II protein production [142]. The sponge *Dysidea avara*, which has a sesquiterpene lactone santonin, has been shown to have antitumor impacts in cancer cell lines; thus, marine drugs may be useful in treatment of cancer [143]. Santonin decreased MMP, enhanced ROS production, caspase-3, and released cyt. c in ALL B-lymphocytes, which caused apoptosis in all B-lymphocytes [143]. Mycothiazole, originally derived from the marine sponge *Petrosaspongia mycofijiensis*, has been shown to have anticarcinoma impacts in cancerous cells via inhibiting hypoxic HIF-1 signaling and causing mitochondrial dysfunction in human breast tumor T47D cells [144]. Mycothiazole has been shown to significantly block mitochondrial respiration at complex I [144]. A dipeptide Cyclo(-Pro-Tyr) (DP) isolated from marine sponge, increased the rate of Bax/Bcl-2 and caspase-3, had negative effects on MMP, stimulated the release of cyt. c, and enhanced the production of ROS in HepG2 cell lines [145]. DP, derived from the marine sponge *Callyspongia fistularis*, has demonstrated an apoptotic role in the destruction of liver cancer HepG2 cell lines and may be a promising medication for the treatment of hepatocellular carcinoma [145]. 

Table 2 summarizes various anticancer marine compounds that interfere with the mitochondrial-related pathways.

Briefly, biologically active secondary metabolites present in marine organisms show a wide range of antineoplastic activities. Marine-derived natural products act by diminishing cell viability, proliferation, promotion of ER stress, apoptosis, and ROS production by interfering with various cell signaling pathways in cancer.

## 5. Conclusion, Challenges, and Future Perspectives

**Figure 6:**
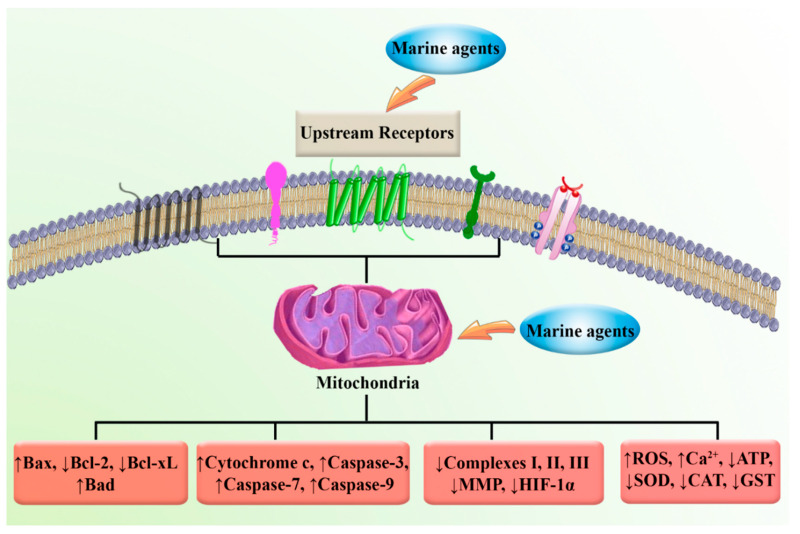
Targeting mitochondria by marine compounds in cancer.

Various dysregulated pathways can influence the growth, proliferation, invasion, and migration of cancer cells. In this line, mitochondria have received attention due to playing several important roles in growth, survival, and in cellular death. These roles and significant effects have made mitochondria and their related signaling pathways key points in preventing progression and aiding in the treatment of cancer. Targeting mitochondrial metabolism-related enzymes, including IDH1, IDH2, SDH, and fumarate hydratase, interfering with PI3K/PTEN/Akt, HIF-1α, WNT, p53, and angiogenesis-related signaling, interfering with mitochondrial metabolism, cellular redox status, increasing the production of ROS, reducing the mitochondrial Ca^2+^ retention, and MMP are some of the most important pathways related to mitochondria that can affect cancer. Several studies have shown the promising advantages of targeting pathways related to mitochondria to inhibit the growth and proliferation of different types of cancer cells. Marine-derived natural products are promising multi-targeted agents that can affect mitochondria signaling pathways. These products act as mediators to suppress cancer cell growth, proliferation, and angiogenesis with fewer side effects (Figure 6). Consequently, these compounds are suitable, promising options for the treatment of cancer through mitochondria.

Mitochondria were shown to be affected by several cytotoxic agents. However, it is often not the primary, but secondary and rather unspecific target that is affected due to the induction of other apoptosis-/cell death-related processes. Pan-Assay Interference Compounds (PAINS) are a recently discovered class of compounds that oppose the “target selectivity” point of view. Such compounds can affect intracellular signaling pathways in a non-specific way, and have been identified as a threat to the bioactivity of natural compounds [146]. PAINS-type behavior causes membrane disruption by decreasing the membrane dipole potential, which affects intracellular signaling pathways. The cell-based data of several natural products are likely to indicate such effects which may not be caused by specific binding to therapeutic targets [147]. This non-specific/specific targeting of mitochondria by marine compounds should be carefully considered in future studies. 

Despite the effectiveness of marine agents, these compounds are often limited by poor bioavailability, low solubility, instability, and low selectivity, which restricts associated therapeutic uses in cancer. Growing clinical studies have demonstrated that marine natural products are encountered with chemical degradation and rapid metabolism and are absorbed with lower efficiency. The aforementioned pharmacokinetic limitations have been attenuated with nanotechnology to enhance bioavailability and anticancer properties of these compounds [4]. However, some clinical trials are in progression to evaluate the clinical potential and efficacy of marine compounds. For instance, the marine compound astaxanthin has shown potential cardioprotective [148] and immunomodulatory [149] effects. It also shows health benefits in overweight/obese adults [150,151], and cosmetic applications [152]. In a double-blind randomized controlled trial, the marine compound fucoidan improved disease control rate in patients with metastatic colorectal cancer [153]. Fucoidan also showed anticancer effects through regulating the expression of microRNAs [154]. In a randomized trial, sodium alginate was found to prevent radiation-induced esophagitis in advanced NSCLC [155]. Several new marine compounds are also being evaluated in patients with solid tumors [156,157,158,159,160].

This comprehensive and critical review underscores the importance of targeting malignancies by multi-targeted marine agents passing through the modulation of mitochondria function. A future area of research should focus more on developing well-controlled clinical trials to assess anticancer potentials of marine agents to overcome the toxicity and drug-resistance of present drugs in the prevention, treatment, and management of cancer.

## Figures and Tables

**Table 1:** Synthetic and plant-derived natural compounds combating cancers through different mitochondria-related signaling pathway.

Compound(s)	Source(s)/Class	In Vitro/In Vivo Models	Mechanisms & Outcomes	References
SSCC	Synthetic	Breast cancer cells (MCF-7, BT-20)	↓Cell size; ↑ROS; ↓MMP; ↑cyt. c	[80]
CS-PAC-AgNPs	Synthetic	Colorectal carcinoma cells (HT-29)	↓Cell proliferation ↑caspase-3; ↑caspase-9; ↓Bcl-2; ↓Bcl-xL; ↑cyt. c	[74]
PD-LPs	Synthetic	Hepatocellular carcinoma cells (SMMC-7721)	↑Caspase-3; ↑Bax; ↓Bcl-2	[73]
Gemcitabine and AgNTs	Synthetic	Glioma cells (U87)	↑ROS; ↓MMP; ↑cyt. c	[81]
Celecoxib and dolastatin 15	Synthetic + natural	Sprague-Dawley rats	↓Bcl-2; ↑caspase-3; ↑caspase-9; ↑cyt. c; ↑p53; ↓MMP	[82]
Ganoleuconin O	Terpenoid	Hepatocellular carcinoma (Huh7.5)	↑Bax; ↑caspase-9; ↑p53; ↑cyt. c; ↓ATP; ↓Bcl-2	[77]
Betulinic acid	Terpenoid	Cervical carcinoma cells (HeLa); Renal cell carcinoma (PC12, and ACHN)	↑Bax; ↑caspase-3; ↑caspase-9; ↑ROS; ↓Bcl-2	[77]
Skimmiarepin A and C	Terpenoid	Breast cancer cells (T47D)	↓HIF-1; ↓complex I	[78]
Ursolic acid	Terpenoid	Breast cancer cells (MCF-7)	↓Cell proliferation; ↓MMP; ↑ROS	[83]
PhCsNPs	Phenolic compound	Oral squamous carcinoma cells (KB)	↑ROS; ↓MMP; ↑cyt. c; ↑Bax; ↑caspase-3; ↑caspase-9; ↓Bcl-2	[84]
Curcumin	Phenolic compound	Hepatocellular carcinoma cells (HepG2)	↑Bax; ↑caspase-3; ↑cyt. c; ↓Bcl-2	[79]
Isoquercitrin	Phenolic compound	Breast cancer cells (MDA-MB-231)	↓MMP; ↓Bcl-2; ↑Bax	[77]

Abbreviations: AIF, apoptosis-inducing factor; ATP, adenosine triphosphate; Bax, Bcl-2-associated X protein; Bcl-2, B-cell lymphoma 2; Bcl-xL, B-cell lymphoma-extra-large; cyt. c, cytochrome c; HIF-1, hypoxia-inducible factor-1; MMP, mitochondrial membrane potential; MMPs, matrix metalloproteinases; NSCLC, non-small cell lung cancer; p53, tumor protein P53; ROS, reactive oxygen species.

**Table 2:** Marine compounds combating cancer through mitochondrial pathway.

Compound(s)	Source(s)	In Vitro/In Vivo Models	Mechanisms & Outcomes	References
Oxalicumone A	Fungus *Penicillium oxalicum*	Cervical adenocarcinoma cells (L-02)	↓ATP; ↑MPTP; ↑Size of mitochondria; ↓matrix density	[88]
Hierridin B	Marine cyanobacterium *Cyanobium* sp.	Colorectal carcinoma cells (HT-29)	↓VDAC1; ↑Ca^2+^; ↑cyt. c	[89]
DDSD	Brown marine algae *Stoechospermum marginatum*	Mouse melanoma cells (B16F10)	↑ROS; ↑cyt. c; ↓Bcl-2/Bax	[90]
Fucoidan	Brown algae	Breast cancer cells (MCF-7 MCF-7, MDA-MB-231); Hepatocellular carcinoma cells (SMMC-7721)	↓Bcl-2; ↑Bax; ↑cyt. c; ↑Bad; ↑ROS	[91,92]
Fucosterol	Brown algae	Cervical carcinoma cells (HeLa); Ovarian cancer cells (ES2, OV90)	↑ROS; ↑caspase-3; ↑caspase-9; ↑cyt. c; ↓MMP	[17,94]
Laminarin	Brown algae	Ovarian cancer cells (ES2, OV90); Colon cancer cells (LOVO)	↓MMP; ↑ROS; ↑Ca^2+^; ↑cyt. c; ↑DNA destruction; ↑caspase-3; ↑Bax	[95]
Astaxanthin	Microalgae *Chlorella zofingiensis*, *Chlorococcum* sp., red yeast *Phaffia rhodozyma*, and the marine *Agrobacterium aurantiacum*	Glioblastoma multiforme cells	↑Bax; ↑Bad; ↓Bcl-2	[118]
Astaxanthin Nanoemulsion		Murine fibroblast cells (L929, NIH3T3)	↑ROS	[118]
9′-*cis*-neoxanthin and fucoxanthin	Green leafy vegetables, brown algae	Colon cancer cells (HL-60)	↑Bax; ↑cyt. c; ↓ATP; ↑AIF	[113,114,115]
Caulerpin	Marine green algae *Caulerpa cylindracea*	Leukemia cells (THP-1)	↓Complex II	[100]
Lagunamide A	Marine cyanobacterium *Lyngbya majuscule*	NSCLC (A549); Cervical carcinoma cells (HeLa); Hepatocellular carcinoma cell (HepG2); Colorectal carcinoma cells (HCT116); Osteosarcoma cells (U2OS)	↓Cell proliferation; ↑ROS; ↓MMP; ↑caspase-3; ↓Bcl-2; ↓Bcl-xL; ↑Bax	[102]
MSP-4-peptide	Fish Nile tilapia (*Oreochromis niloticus*)	Osteosarcoma cells (MG63)	↓Bcl-2; ↑Bax; ↑Bid; ↑cyt. c; ↑caspase-3; ↑caspase-9	[103]
18B-15-3	Marine fungus *Aspergillus* sp.	Pancreatic cancer cells (PANC-1)	↓MMP; ↓complex III; ↓intake of oxygen	[138]
EI-SP	Marine algae *Enteromorpha intestinalis*	Hepatocellular carcinoma cells (HepG2)	↓Bcl-2; ↑Bax; ↑cyt. c; ↑caspase-9	[98]
Thyrsiferol	Red algae *Laurencia thyrsifera* J.Agardh	Breast cancer cells (T47D)	↓Complex I; ↓HIF-1; ↓oxygen utilization	[99]
Conus textile	Marine cone snails	Glioma cells (U87MG)	↑Caspase-3; ↑caspase-9; ↑cyt. c; ↑ROS; ↑Bax/Bcl-2	[104]
Phallusia nigra	Marine tunicate	Albino\Wistar rats	↑ROS; ↑cyt. c	[106]
Aplidin	Marine tunicate *Aplidium albicans*	leukemia lymphoma models	↑ROS; ↓ATP; ↓MMP	[107]
Holothuria parva and Haliclona oculata	Sea cucumber	Sprague-Dawley rats	↑ROS; ↑cyt. c; ↑caspase-3	[139]
Kalkitoxin	Marine cyanobacterium *Lyngbya majuscula*	Breast cancer cells (T47D)	↓Complex I; ↓HIF-1	[108]
MDTFC	Soft coral	Leukemia cells (THP-1)	↑Caspase-3; caspase-9; ↑Bax/Bcl-2; ↑cyt. c; ↓MMP	[109]
ECHC	Coral *Acropora formosa*	NSCLC (A549)	↑ROS; ↑cyt. c; ↑Bax; ↓Bcl-2; ↓TNF-α; ↓IL-8; ↓MMP2; ↓MMP9	[110]
Turbo coronatus	Marine mollusk	Ovarian cancer cells (*EOC)*	↑ROS; ↑cyt. c; ↑caspase-3; ↓MMP	[111]
Mansouramycin C	Marine streptomycete	NSCLC (A549)	↑ROS; ↓ATP; ↓MMP; ↑mitochondrial swelling	[112]
Arca inflata	Bivalve mollusk	Colorectal cancer cells	↑ROS; ↑Ca^2+^; ↑cyt. c; ↓MMP; ↑caspase-3; ↓cell growth	[119]
CS5931	Sea squirt *Ciona savignyi*	Colorectal carcinoma cells (HCT116)	↑Caspase-3; ↑caspase-9; ↑cyt. c; ↑Bax; ↓MMP	[120]
Lamellarin D	Marine mollusk *Lamellaria*	Leukemia cells (p388)	↑Bax; ↑caspase-3; ↑caspase-9; ↓Bcl-2	[121,122,123]
Irciniastatin A	Marine sponge *Ircinia ramosa*	Pancreatic cancer cells (PANC-1)	↑JNK; ↑p38	[16]
GLP	Green alga *Codium decorticatum*	Breast cancer cells (MDA-MB-231)	↑ROS; ↑cyt c; ↑caspase-3; ↑caspase-9; ↓MMP	[125]
Avicennia marina combined with Ag NPs	Marine mangrove plant	NSCLC (A549)	↑ROS; ↓MMP	[126]
Pterocellin A	Marine bryozoan *Pterocella vesiculosa*	Cervical carcinoma cells (HeLa)	↑Caspase-3; ↑nucleus condensation	[127]
Chlorella sorokiniana	Green algae	NSCLC	↑Caspase-3; ↑caspase-9; ↑Bax; ↓MMP; ↑cyt. c; ↓Bcl-2	[101]
Heteronemin	Marine spong *Hippospongia* sp.	Hepatocellular carcinoma cells	↑ROS; ↑Bax; ↑SOD2; ↑caspase 8; ↓Bcl-2; ↓SOD1	[129]
Sphaerodactylomelo	Red alga *Sphaerococcus coronopifolius*	Breast cancer cells (MCF-7)	↑ROS; ↑caspase-3; caspase-9; ↑H_2_O_2_; ↓MMP; ↑cyt. c; ↓Bcl-2	[130]
Ilimaquinone	Marine sponges *Halichondria* sp.	Breast cancer cells (MCF-7, MDA-MB-231)	↑ROS; ↑caspase-3; ↑caspase-9; ↓MMP	[131]
Manzamine A	Sponges *Haliclona* sp., *Xestospongia* sp., and *Pellina* sp.	Colorectal carcinoma cells (HCT116)	↑Caspase-3; ↑caspase-7; ↓MMP; ↓Bcl-2; ↓Bcl-xL	[136]
Aplysinopsins	Genera of sponges and scleractinian corals	Leukemia cells (K562)	↓Cell proliferation; ↓Bcl-2; ↓MMP; ↓oxygen utilization;	[137]
Lipophilic 2,5-disubstituted pyrroles	Marine sponge *Mycale* sp.	Breast cancer cells (T47D)	↓HIF-1; ↓complex I; ↑ROS	[140]
Urupocidin A	Marine sponges	Prostate cancer cells (22Rv1, LNCaP and MRC-9)	↑ROS; ↓MMP; ↓Bcl-2	[87]
Papuamine	Marine sponge *Neopetrosia chaliniformis*	Breast cancer cells (MCF-7)	↓MMP; ↑cyt. c; ↑Bax	[141]
10-acetylirciformonin B	Marine sponge	Acute myeloid leukemia cells (HL 60)	↑ROS; ↑cyt. c; ↑Bax; ↓Bcl-2; ↓Bcl-xL	[142]
α-Santonin	Sponge *Dysidea avara*	ALL B-lymphocytes	↑ROS; ↑caspase-3; ↓MMP; ↑cyt. c	[143]
DP	Marine sponge *Callyspongia fistularis*	Hepatocellular carcinoma cell (HepG2)	↑Bax/Bcl-2; ↑caspase-3; ↓MMP; ↑ROS; ↑cyt. c	[145]

Abbreviations: AIF, apoptosis-inducing factor; ATP, adenosine triphosphate; BAD, Bcl-2 associated agonist of cell death; Bax, Bcl-2-associated X protein; Bcl-2, B-cell lymphoma 2; Bcl-xL, B-cell lymphoma-extra-large; COX-2, cyclooxygenase-2; cyt. c, cytochrome c; DNA, deoxyribonucleic acid; DP: dipeptide Cyclo(-Pro-Tyr), GST, glutathione S-transferase; H_2_O_2_, hydrogen peroxide; HIF-1α, hypoxia-inducible factor 1α; IL-8, interleukin 8; JNK, c-Jun NH2-terminal kinase; MMP, mitochondrial membrane potential; MMPs, matrix metalloproteinases; MPTP, mitochondrial permeability transition pore; NSCLC, non-small cell lung cancer; ROS, reactive oxygen species; SOD1, superoxide dismutase; TNF-α, tumor necrosis factor-α; VDAC1, voltage-dependent anion channel-1.
